# Supplementary material for: Development of BCR-ABL1 Transgenic Zebrafish Model Reproducing Chronic Myeloid Leukemia (CML) Like-Disease and Providing a New Insight into CML Mechanisms
Source: Cells. 2021 Feb 19;10(2):445. doi: 10.3390/cells10020445 (PMC7922348; doi:10.3390/cells10020445)
Supplement: Supplementary file 1 [file cells-10-00445-s001.pdf]

Supplementary Figures

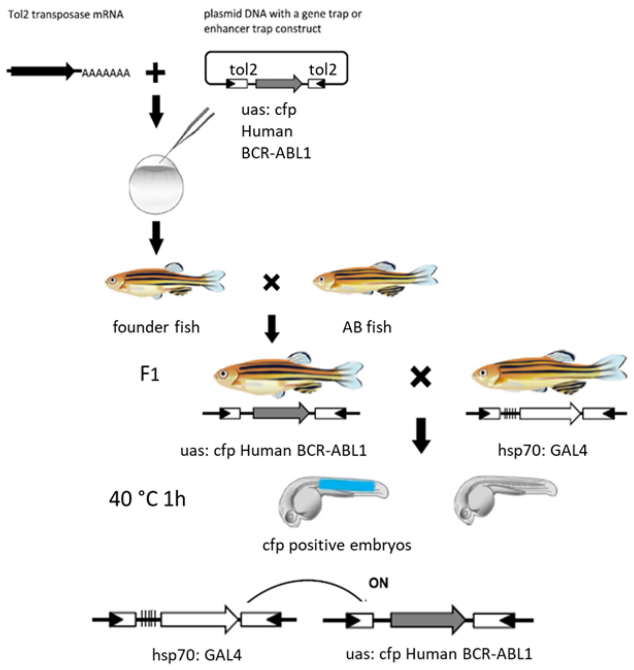

Figure 1. Schematic representation of strategy used to obtain the transgenic line Tg BCR-ABL1pUAS/hsp70-Gal4.

A **Percentage of identity between human and zebrafish**

	**Abl1**	**Bcr**
	Danio rerio	*Danio rerio*
Homo sapiens	**72 %**	**76 %**

B

Conservation of kinase domain of Abl1 zebrafish protein

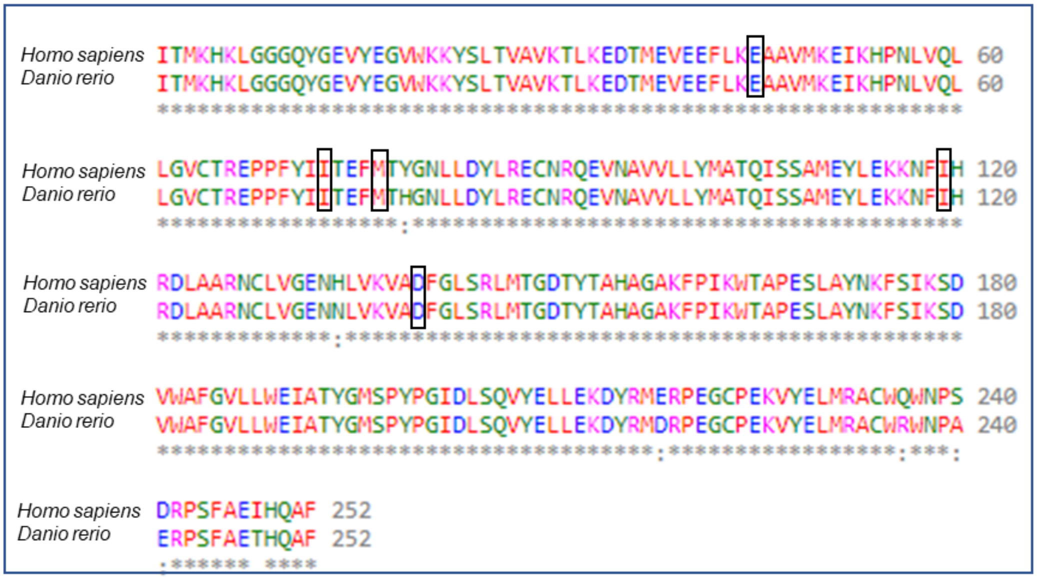

Figure 2. (**A**) Multiple sequences alignment was performed using the MUSCLE algorithm. Accession numbers of zebrafish proteins Bcr (XP_002665983), Abl1 (XP_005172104) and human proteins accession number BCR (NP_004318) and ABL1

(NP_005148) (**B**) Comparison of kinase domain sequence of human and zebrafish Abl1. The kinase domain is highly conserved (97%) and all the amino acids (black box) that are involved in bonds with tyrosin kinase inhibitors are preserved.

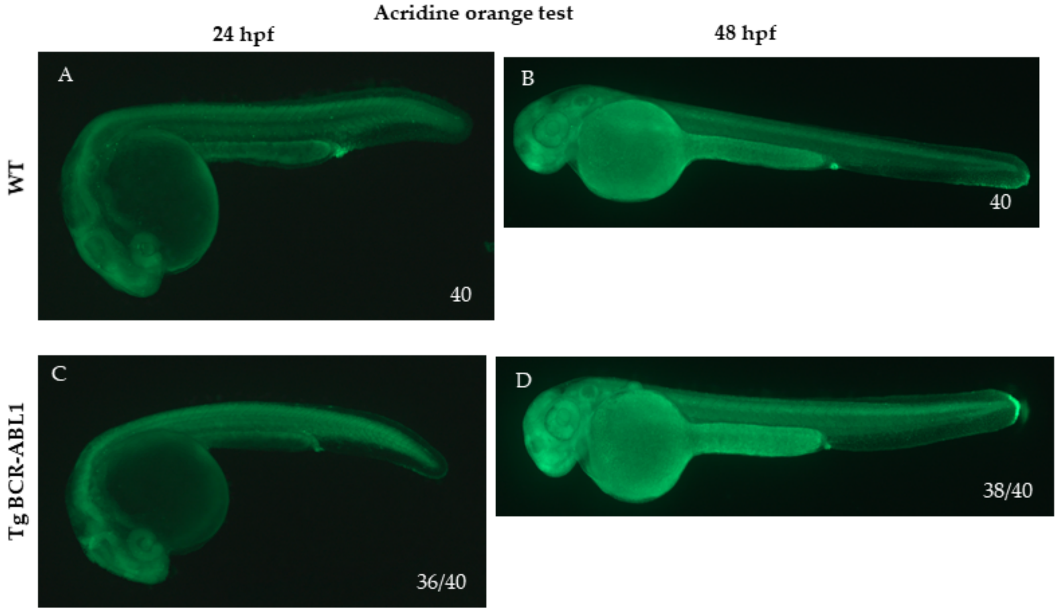

Figure 3. Acridine orange assay. Embryos were tested for apoptosis by acridine orange assay. Wild Type (**A**) and (**B**) and Tg BCR-ABL1 (**C**) and (**D**) embryos were analysed respectively at 24 and 48 hpf. The images are representative of one experiment out of three (Magnification 20X). The numbers indicate the observed embryos
